# Styrene Maleic Acid Copolymer-Based Micellar Formation of Temoporfin (SMA@ mTHPC) Behaves as A Nanoprobe for Tumor-Targeted Photodynamic Therapy with A Superior Safety

**DOI:** 10.3390/biomedicines9101493

**Published:** 2021-10-19

**Authors:** Jun Fang, Shanghui Gao, Rayhanul Islam, Hinata Nema, Rina Yanagibashi, Niho Yoneda, Natsumi Watanabe, Yuki Yasuda, Naoki Nuita, Jian-Rong Zhou, Kazumi Yokomizo

**Affiliations:** Faculty of Pharmaceutical Sciences, Sojo University, Ikeda 4-22-1, Kumamoto 860-0082, Japan; gaoshanghui94@gmail.com (S.G.); rayhanulislam88@gmail.com (R.I.); g1651090@m.sojo-u.ac.jp (H.N.); g1651124@m.sojo-u.ac.jp (R.Y.); g1651133@m.ph.sojo-u.ac.jp (N.Y.); g1651134@m.sojo-u.ac.jp (N.W.); g1751126@m.sojo-u.ac.jp (Y.Y.); g1751093@m.sojo-u.ac.jp (N.N.); zhoujr@ph.sojo-u.ac.jp (J.-R.Z.); yoko0514@ph.sojo-u.ac.jp (K.Y.)

**Keywords:** EPR effect, polymeric micelles, PDT nanoprobe, tumor targeting, temoporfin

## Abstract

Tumor-targeted photodynamic therapy (PDT) using polymeric photosensitizers is a promising anticancer therapeutic strategy. Previously, we developed several polymeric nanoprobes for PDT using different polymers and PDT agents. In the study, we synthesized a styrene maleic acid copolymer (SMA) micelle encapsulating temoporfin (mTHPC) that is a clinically used PDT drug, SMA@mTHPC, with a hydrodynamic size of 98 nm, which showed high water solubility. SMA@mTHPC maintained stable micelle formation in physiological aqueous solutions including serum; however, the micelles could be disrupted in the presence of detergent (e.g., Tween 20) as well as lecithin, the major component of cell membrane, suggesting micelles will be destroyed and free mTHPC will be released during intracellular uptake. SMA@mTHPC showed a pH-dependent release profile, for which a constant release of ≈20% per day was found at pH 7.4, and much more release occurred at acidic pH (e.g., 6.5, 5.5), suggesting extensive release of free mTHPC could occur in the weak acidic environment of a tumor and further during internalization into tumor cells. In vitro cytotoxicity assay showed a lower cytotoxicity of SMA@mTHPC than free mTHPC; however, similar in vivo antitumor effects were observed by both SMA@mTHPC and free THPC. More importantly, severe side effects (e.g., body weight loss, death of the mice) were found during free mTHPC treatment, whereas no apparent side effects were observed for SMA@mTHPC. The superior safety profile of SMA@mTHPC was mostly due to its micelle formation and the enhanced permeability and retention (EPR) effect-based tumor accumulation, as well as the tumor environment-responsive release properties. These findings suggested SMA@mTHPC may become a good candidate drug for targeted PDT with high safety.

## 1. Introduction

Photodynamic therapy is a less invasive therapeutic strategy for cancer, which utilizes photosensitizers (PS) followed by light irradiation [1,2,3]. Upon light exposure, the PS is excited, and the energy is transferred to molecular oxygen to generate cytotoxic singlet oxygen (^1^O_2_) [3]. ^1^O_2_, as an oxygen free radical, rapidly react with biomolecules, i.e., proteins, DNA, and lipid, inducing oxidative damage and apoptosis of the cells [3,4,5]. Most of the PSs are non-toxic or less toxic agents and they are not harmful without exposure to the light; accordingly, tumor-specific light irradiation will kill cancer cells selectively, without inducing severe side effects to the normal cells, which is an advantage to conventional anticancer chemotherapy. However, conventional PSs are mostly low-molecular weight agents—after systemic administration, they distribute indiscriminately to both tumor tissue and normal tissues, e.g., the skin; ambient light may thus trigger the injury and inflammation of the skin. Actually, in conventional PDT, patients can remain photosensitive for several weeks after treatment, and avoiding excess ambient light is always necessary for patients receiving PDT [4]. In addition, most PSs show poor water-solubility, which hampers their clinical application.

In order for these drawbacks to be overcome, nano-designed PSs have been receiving much attention. Namely, biocompatible polymers, liposome, and antibodies are used to modify PSs resulting in various macromolecular formulations of PSs with sizes of several to several hundred nanometers. The nano-formulation of PSs renders high water solubility of PSs, and more importantly, it could fulfill tumor-targeted PDT effect by taking advantage of the enhance permeability and retention (EPR) effect. EPR effect is a unique phenomenon regarding the behaviors of macromolecules according to the abnormal anatomical and pathophysiological natures of tumor blood vasculature [6,7,8,9,10,11]. Compared to normal vasculature, tumor vasculature shows a large gap between the endothelial cells and exhibits high vascular permeability, as well as defected lymphatic functions, by which macromolecules with molecular weight higher than 40–50 kDa, or molecular size larger than 5–10 nm, will accumulate selectively and remain in tumor tissues for prolonged period of time, whereas they will not penetrate normal blood vessels, thus showing significantly less distribution in normal tissues compared to low molecular weight agents. The EPR effect was first discovered by Maeda and Matsumura in 1986 [6], and now it has been become a well-understood rationale for the design and development of anticancer nanomedicine [7,8,9,10,11]. In our laboratory, on the basis of the EPR effect, we have developed many macromolecular anticancer agents by using biocompatible polymers including polyethylene glycol (PEG), styrene maleic acid copolymer (SMA), and poly(N-(2-hydroxypropyl) methacrylamide) copolymer (HPMA) [7,8,9,10,11,12,13,14,15,16,17,18,19]. Polymer-modified PSs were also investigated, including PEG-conjugated zinc protoporphyrin (ZnPP) (PEG-ZnPP) [19,20], SMA micelles of ZnPP (SMA-ZnPP) [16,17,18], HPMA-conjugated ZnPP (HPMA-ZnPP) [12,14], and HPMA-conjugated pyropheophorbide a (P-PyF) [15], all of which showed tumor-targeting properties and potent PDT effect with high tumor selectivity. Along this line, in this study, we challenged a polymeric micellar formation of a clinically used PDT drug, temoporfin (mTHPC), using SMA copolymer.

mTHPC is the most potent second-generation PS [21], and it is approved in the European Union as a PDT drug for the treatment of squamous cell carcinoma of the head and neck [4]. As with other PSs, administration of mTHPC results in patients becoming highly sensitive to light, which lasts 7 to 15 days, and therefore appropriate light exposure precautions are necessary during this period [4]. mTHPC is water-insoluble and its standard formulation is dissolved in organic solvents, i.e., ethanol, which largely hampers its application. Accordingly, liposomal formulations of mTHPC have been developed showing high water-solubility as well as potent PDT effect [21,22,23,24], suggesting the benefit of nano-design for mTHPC. 

Besides liposomal formulation, polymer micelle is another well-accepted nano-platform, in which amphiphilic polymers are utilized to form micelles in aqueous solutions by self-assembly where hydrophobic drugs are encapsulated in the core of micelles [25,26]. SMA is one such amphiphilic copolymer, containing hydrophobic styrene motif and hydrophilic maleic acid motif. We have successfully developed several SMA micelles of anticancer agents including doxorubicin, pirarubicin, and ZnPP, all of which showed high water solubility and tumor-targeting properties [18,27,28]. In this context, we report here a SMA micelle encapsulating mTHPC (SMA@mTHPC), which showed increased water solubility, potent PDT effect, and superior safety profile compared to native mTHPC.

## 2. Materials and Methods

### 2.1. Chemicals

Poly(styrene-co-maleic anhydride) (an SMA copolymer), with a mean molecular weight of 1600 Da, and mTHPC were purchased from Sigma Chemical Co. (St. Louis, MO, USA). 3-(4,5-Dimethyl-2-thiazolyl)-2,5-diphenyl-2H-tetrazolium bromide (MTT) and 1-ethyl-3-(3-dimethylaminopropyl) carbodiimide hydrochloride (WSC) were purchased from Wako Pure Chemical Industries Ltd. (Osaka, Japan). 2,2,6,6-Tetramethyl-4-piperidone (4-oxo-TEMP) was purchased from Tokyo Chemical Industry (Tokyo, Japan). Other reagents of reagent grade and solvents were purchased from Wako Pure Chemical Industries Ltd. and used without further purification. 

### 2.2. Synthesis of SMA@mTHPC

#### 2.2.1. Hydrolysis and Purification of SMA

The maleic anhydride residue of the SMA copolymer was hydrolyzed to the water-soluble maleic acid form by addition of 1 N NaOH at 50 mg/mL. The solution was heated at 50 °C during stirring for 24 h until a clear solution was obtained. Then, the pH of the solution was adjusted to 7.0 with 1 N HCl, followed by dialysis using a dialysis bag with molecular cut-off of 8000 Da (Wako), and then freeze-drying. 

#### 2.2.2. Preparation of SMA@mTHPC Micelles

SMA@mTHPC micelles were prepared by a similar protocol to that described earlier by us for SMA-ZhPP micelles [18], with some modifications. In brief, hydrolyzed SMA (100 mg) was dissolved in 20 mL deionized water and the pH was adjusted to 5.0 by 1 N HCl, to which 11 mg of mTHPC dissolved in 1 mL DMSO was added dropwise. One hundred milligrams of WSC was then added, and the reaction mixture was stirred at room temperature for 30 min. Then, the pH of reaction solution was adjusted to 11.0 by 1 N NaOH, with further stirring for 1 h. Finally, the pH or the reactant was adjusted to 7.4 by 1N HCl, followed by dialysis against deionized water at 4 °C for 3 days with 3-change of water, and then freeze-drying, in order to obtain the brown powder of SMA@mTHPC (91 mg).

### 2.3. Characterization of P-PyF

#### 2.3.1. Measurement of Particle Size of P-PyF

SMA@mTHPC was dissolved in 0.01 M phosphate-buffered 0.15 M saline (PBS; pH 7.4) at 2.5 mg/mL and was filtered through a 0.2 μm filter. The particle size was measured by dynamic light scattering (ELS-Z2; Otsuka Photal Electronics Co. Ltd., Osaka, Japan). 

#### 2.3.2. Fluorescence Spectroscopy

Fluorescence spectra of SMA@mTHPC, dissolved in different solutions or solvents, were recorded on a spectrophotometer (FP6600, Jasco Corp.,Tokyo, Japan). The sample solution was excited at 420 nm (corresponding to the maximum absorbance of mTHPC), and emission from 600 to 800 nm was recorded. A standard curve for free mTHPC in DMSO was plotted as a reference for quantification of the release of mTHPC from SMA@mTHPC as describe below.

#### 2.3.3. UV–VIS Spectroscopy

UV–VIS spectra of SMA@mTHPC were recorded on a spectrophotometer (V730, Jasco Corp.). mTHPC content was quantified on the basis of analysis of UV–VIS absorption of SMA@mTHPC that was dissolved in DMSO at 420 nm. A standard curve for free mTHPC in DMSO was plotted (inset of Figure S1) as a reference for calculating the loading of mTHPC in SMA@mTHPC.

#### 2.3.4. Release Rate of mTHPC from the SMA@mTHPC Micelles

The release of mTHPC from SMA@mTHPC micelles was measured by a dialysis method. In brief, 5 mg of SMA@mTHPC micelles was dissolved in 1 mL deionized water and placed in sealed dialysis bags (Mw cut-off 8000 Da, Wako). The dialysis bags were submerged in 50 mL tubes (Falcon, BD labware, Franklin Lakes, NJ) containing 25 mL of 0.2 M sodium phosphate buffers of different pH values (i.e., pH 5.5, pH 6.5, and pH 7.4). The dialysis tubes were then incubated at 37 °C in the dark with reciprocal shaking at 1 Hz. The mTHPC released from the dialysis bags were collected at scheduled time intervals and its amount was quantified by recording fluorescence intensity after 10-time dilution by DMSO by using the standard curve of mTHPC.

### 2.4. Detection of ^1^O_2_ Generation by Electron Spin Resonance (ESR) Spectroscopy

SMA@mTHPC was dissolved in PBS at 400 μg/mL (40 μg/mL mTHPC equivalent) with/without 0.1% Tween 20, to which 20 mM 4-oxo-TEMP (spin trapping agent) was added. Samples in a flat quartz cell (Labotec, Tokyo, Japan) were irradiated (25 mW/cm^2^) for the indicated times, by using xenon light source (MAX-303; Asahi Spectra Co. Ltd., Tokyo, Japan) at 400–700 nm. The ESR spectrometer was usually set at a microwave power of 1.0 mW, amplitude of 100 kHz, and field modulation width of 0.1 mT.

### 2.5. In Vitro Cytotoxicity Assay

Mouse colon cancer C26 cells and African green monkey kidney cells (CCL-81) were maintained in RPMI-1640 medium (Wako), supplemented with 10% fetal calf serum (Nichirei Biosciences Inc., Tokyo, Japan) under 5% CO_2_/air at 37 °C. Cells were seeded in 96-well plates at 5000 cells per well and preincubated for 24 h. SMA@mTHPC was then added at different concentrations, followed by irradiation with fluorescent blue light that had peak emission at 420 nm (1.0 J/cm^2^) (TL-D; Philips, Eindhoven, the Netherlands) at 24 h after addition of SMA@mTHPC. After further 24 h of culture, the MTT assay was carried out to quantify viable cells. In some experiments, the dark cytotoxicity of SMA@mTHPC without light irradiation was carried out, in which MTT assay was performed at 48 h after SMA@mTHPC administration. 

### 2.6. Intracellular Uptake of SMA@mTHPC

C26 cells were seeded in 12-well plates at 3 × 10^5^ cells per well and preincubated for 24 h. Free mTHPC or SMA@mTHPC was then added at 2 μg/mL. After the desired time, the cells were harvested and collected. After being washed thrice with PBS, the internalized mTHPC were extracted by using ethanol under sonication (30 W, 30 s, UP50H homogenizer, Hielscher Ultrasonics GmbH, Teltow, Germany) on ice, and the supernatant subsequently obtained after centrifugation (13,000 rpm, 15 min) was subjected to fluorescence spectroscopy (excitation at 420 nm, emission at 590 nm). The amount of mTHPC was then calculated by using the standard curve of mTHPC (Figure S2). In some experiments, the culture medium of pH 5.5 was used to investigate the uptake of SMA@mTHPC in different pH conditions.

### 2.7. In Vivo Tissue Distribution of SMA@mTHPC

Male ddY mice used in this study were 6 weeks old and obtained from SLC Inc., Shizuoka, Japan. Mouse sarcoma S180 cells (2 × 10^6^ cells) that had been grown in peritoneal cavity of ddY mice as ascetic form were implanted subcutaneously (s.c.) in the dorsal skin of ddY mice in order to establish a mouse S180 solid tumor model. All animals were maintained under standard conditions and fed water and murine chow ad libitum. All animal experiments were approved by the Animal Ethics Committees of Sojo University (no. 2020-P-009, approved on 1 April 2020) and were carried out according to the Guidelines of the Laboratory Protocol of Animal Handling, Sojo University. 

At 10–12 days after tumor inoculation when the diameters of the tumor reached approximately 10 mm, 5 mg/kg (mTHPC equivalent) of SMA@mTHPC dissolved in physiological saline was injected intravenously (i.v.). At 24 h after injection, the mice were sacrificed. After perfusion with physiological saline, tumors as well as normal tissues, e.g., liver, spleen, and kidney etc., were then dissected and weighed, and DMSO (1 mL/100 mg of tissue) was added. Tissues were then homogenized and after centrifugation (12,000 × *g*, 25 °C, 10 min), and mTHPC extracted in the supernatant was quantified by fluorescence intensity (Ex. at 420 nm, Em. at 590 nm) by using a standard curve of mTHPC (Figure S2).

In some experiments, the collected tumors as well as normal tissues (i.e., the liver) were subjected to ex vivo imaging using IVIS XR (Caliper Life Science, Hopkinton, MA, USA).

### 2.8. Comparison of the In Vivo Toxicity of SMA@mTHPC with Native mTHPC

The mouse S180 tumor model described above was used in this study. At 7–10 days after tumor inoculation when the diameters of tumors reached approximately 8–10 mm, SMA@mTHPC dissolved in physiological saline was injected intraperitoneally (i.p.) at a concentration of 10 mg/kg (mTHPC equivalent). Native mTHPC that was dissolved in DMSO was administered i.p. at the same concentration (10 mg/kg). To some of the mice receiving SMA@mTHPC or mTHPC, irradiation to the tumor area was carried out by xenon light (MAX-303; Asahi Spectra) at 400–700 nm for 5 min (27 J/cm^2^) at 24 h after injection of SMA@mTHPC or mTHPC. The conditions and survival of the mice were monitored regularly.

In a separate study, SMA@mTHPC or mTHPC was injected i.v. at 20 mg/kg, in which mTHPC was first dissolved in DMSO and further diluted 10 times by physiological saline to indicated concentration.

### 2.9. In Vivo Antitumor Activity of SMA@mTHPC

The mouse S180 tumor model described above was used in this study. At 7–10 days after tumor inoculation when the diameters of tumors reached approximately 8–10 mm, SMA@mTHPC or mTHPC (10 mg/kg, mTHPC equivalent) was administered i.v. At 24 and 48 h after injection, the tumor was irradiated by xenon light (MAX-303; Asahi Spectra) at 400–700 nm for 5 min (27 J/cm^2^). Our previous studies verified that xenon light source is an efficient tool for PDT that could cover most of the absorptions of PS with high intensity and low cost [14,15]. The width (W) and length (L) of the tumors, as well as the body weight of mice, were measured every 2–3 days during the study period, and tumor volume (mm^3^) was calculated as (W^2^ × L)/2. The survival rate of animals was also recorded.

### 2.10. Statistical Analyses

All data were expressed as means ± SD. Data were analyzed by using ANOVA followed by the Bonferroni *t*-test. A difference was considered statistically significant when *p* < 0.05.

## 3. Results

### 3.1. Synthesis and Characterization of P-PyF

**Figure 1:**
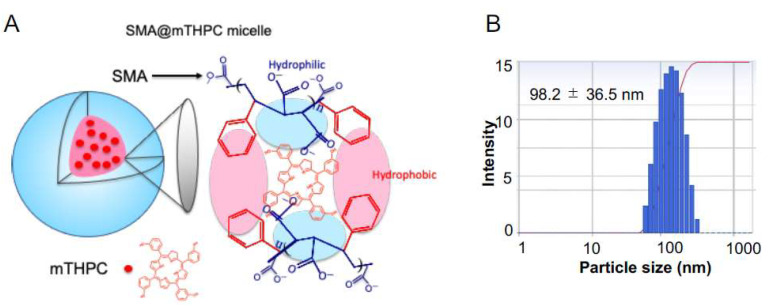
(**A**) Diagrammatic illustration of the micelle structure of SMA@mTHPC and (**B**) the hydrodynamic size of SMA@mTHPC in aqueous solution determined by dynamic light scatter (DLS).

As shown in Figure 1A, SMA@mTHPC micelles form by self-assembly with the hydrophobic core encapsulating mTHPC and hydrophilic outer phase of maleic acid. SMA@mTHPC shows good water-solubility, and a clear solution was found at 20 mg/mL in PBS without precipitate after centrifugation (12,000 rpm, 1 min) (Figure S3). In aqueous solution, it exhibits a molecular size of 98 nm (Figure 1B), indicating the formation of micelles. 

**Figure 2:**
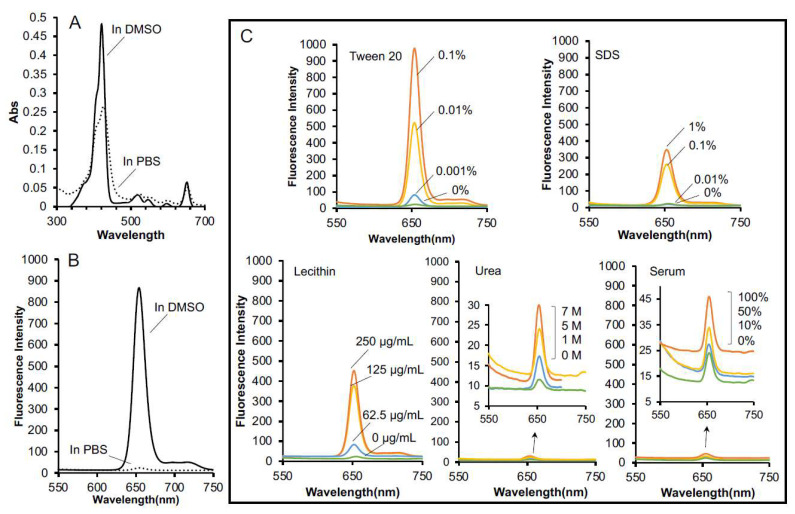
Characterization of the micelle formation of SMA@mTHPC. The UV–VIS spectra (**A**) and fluorescence spectra (**B**) of SMA@mTHPC in PBS as well as in DMSO were measured. The fluorescence spectra of SMA@mTHPC in the presence of detergent (Tween 20, SDS), the cell membrane component (lecithin), urea, and serum are shown in (**C**). See text for details.

The UV–VIS spectrum of SMA@mTHPC in DMSO was similar to that of native mTHPC (Figure S1), but a decreased and shifted spectrum was found when it was dissolved in PBS (Figure 2A), suggesting the formation change in different solvents, i.e., micelles was formed in PBS, but the micelles were disrupted in organic solvent DMSO resulting in the similar UV–VIS spectrum to free mTHPC. This finding also in part supported the micelle formation of SMA@mTHPC in aqueous solutions. By using the standard curve (concentration vs. UV–VIS absorption) of mTHPC, the mTHPC loading in SMA@mTHPC was calculated as 10% (Figure S1).

The micelle formation of SMA@mTHPC in aqueous solution was further confirmed by detection of fluorescence. When mTHPC is encapsulated in the core of polymer micelle, aggregation of mTHPC molecules will occur, resulting in intense intermolecular π–π stacking interactions, consequently leading to fluorescence quenching, i.e., the decrease of fluorescence intensity [29]. As shown in Figure 2B, strong fluorescence was observed from SMA@mTHPC when it was dissolved in DMSO, in which the micelle formation was completely disrupted; however, the fluorescence of SMA@mTHPC in PBS was markedly quenched and was almost indetectable. Fluorescence quenching could also be liberated in the presence of Tween 20 and sodium dodecyl sulfate (SDS), which are surfactants disrupting the micelle self-assembly (Figure 2C), but urea did not affect fluorescence quenching (Figure 2C). These findings suggested hydrophobic interactions, but not hydrogen bond, may be involved in the micelle formation of SMA@mTHPC. More importantly, improvement of fluorescence was also observed in the presence of lecithin, the major component of cell membrane, but no increase of fluorescence was found in the presence of serum (Figure 2C), which indicated that SMA@mTHPC could behave as micelles stably in circulation; however, when it is taken up by cells, micelles will be disrupted to release free mTHPC rapidly.

### 3.2. Release of Free mTHPC from SMA@mTHPC

**Figure 3:**
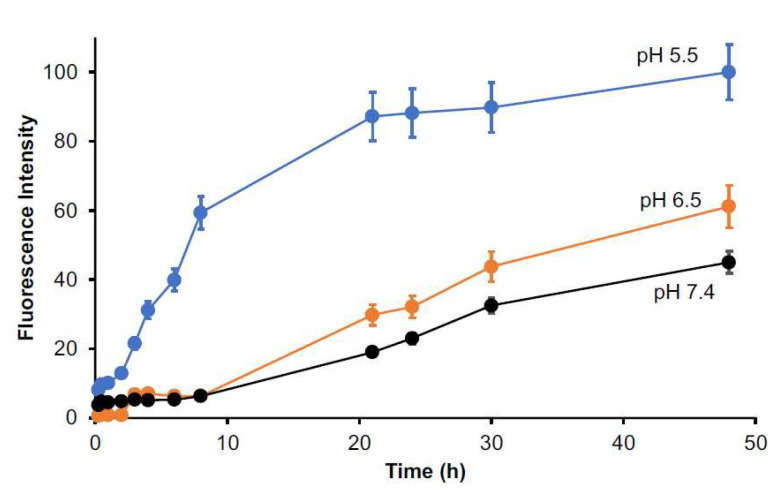
Release profile of SMA@mTHPC. SMA@mTHPC was dissolved in sodium phosphate buffer of different pH values and sealed in dialysis bags. After the indicated incubation time at 37 °C, the mTHPC released from the dialysis bags were measured and quantified by recording fluorescence intensity. A constant in vitro release rate of ≈20% per day was observed at neutral pH (7.4), whereas higher release was found at weak acidic pH (6.5, 5.5). Data are mean ± SD, *n* = 4. See text for details.

Release of free drug is a key issue for polymeric micellar drugs to fulfill their pharmacological effects. We thus investigated the release profiles of SMA@mTHPC in different conditions. In buffer solution of neutral pH (7.4), a constant release of free mTHPC, i.e., ≈20% per day, was observed (Figure 3). 

The release of mTHPC was largely increased at acidic pH; the release rate reached 50% after 48 h incubation at pH 6.5, and it further reached 90–100% at pH 5.5 (Figure 3). Given that tumors always show slight acidic pH (6.0–7.0) [30], tumor-specific release of free mTHPC could be anticipated for SMA@mTHPC.

### 3.3. Generation of ^1^O_2_ from SMA@mTHPC under Light Irradiation

**Figure 4:**
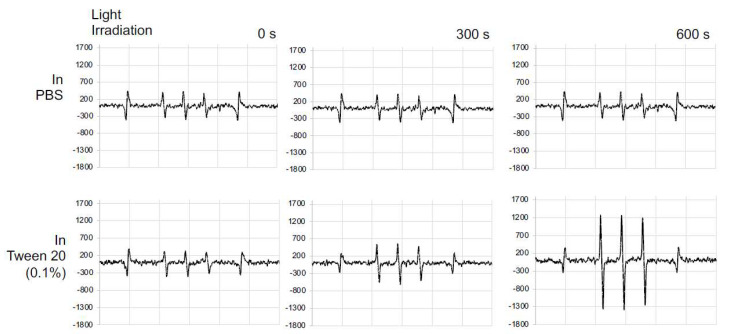
ESR measurement for singlet oxygen (^1^O_2_) generation from SMA@mTHPC. SMA@mTHPC was dissolved in PBS in the absence or presence of 0.1% Tween 20, and light irradiation (25 mW/cm^2^) was carried out using xenon light of 400–700 nm, for the indicated times. ^1^O_2_ generated was captured by 4-oxo-TEMP, and triplet 4-oxo-TEMPO signal due to ^1^O_2_ was detected by ESR spectra. See text for details.

To elucidate the efficacy of SMA@mTHPC to produce ^1^O_2_, we measured the ^1^O_2_ generation using ESR. As shown in Figure 4, the ^1^O_2_ generation from SMA@mTHPC was found negligible or very little in PBS; however, strong signal of ^1^O_2_ was detected in an irradiation-dependent manner when Tween 20 was added into the solution (Figure 4). These findings were consistent with results of fluorescence quenching shown in Figure 2C, indicating that the micelle formation of SMA@mTHPC in aqueous solution also suppressed the generation of ^1^O_2_, with disruption of micelle being necessary for SMA@mTHPC to achieve PDT effect.

### 3.4. In Vitro Cytotoxicity of SMA@mTHPC

On the basis of the findings of ^1^O_2_ generation described in Figure 4, we investigated the PDT effect of SMA@mTHPC in vitro by using a fluorescence blue light source that fits to the maximal absorbance of mTHPC.

**Figure 5:**
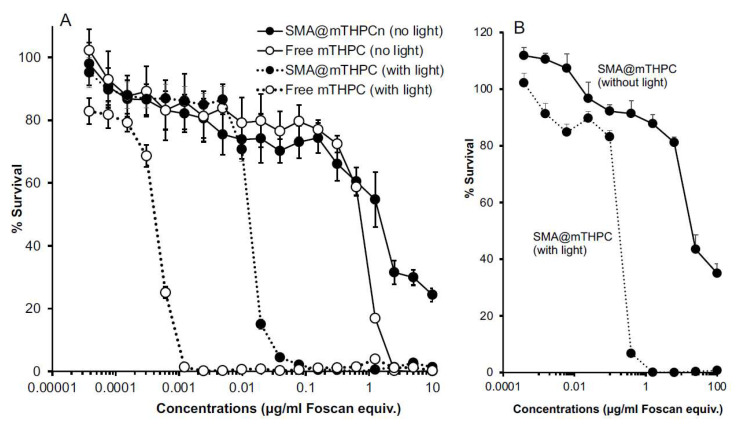
In vitro dark cytotoxicity and photocytotoxicity (PDT) of SMA@mTHPC in mouse colon cancer C26 cells (**A**) and African green monkey kidney cells (CCL-81) (**B**). Cells (5000/well) were seeded in a 96-well plate; after 24 h pre-incubation, different concentrations of SMA@mTHPC were added, and after further 24 h incubation, the viability of cells was measured by MTT assay. Photocytotoxicity was examined by irradiating the cells with light (blue light of 420 nm, 1 J/cm^2^) at 24 h after addition of SMA@mTHPC. Data are mean ± SD, *n* = 6–8. See text for details.

As shown in Figure 5A, in cultured C26 colon cancer cells, SMA@mTHPC alone (no light irradiation) induced the cell death with an IC_50_ of 2 μg/mL that was slightly lower than the cytotoxicity of free mTHPC (IC_50_ of 1 μg/mL). However, after irradiation using blue light source (1.0 J/cm^2^), cytotoxicities of both free mTHPC and SMA@mTHPC were remarkably increased (more than 100-fold), and the IC_50_ of PDT using SMA@mTHPC and free mTHPC were 0.015 μg/mL and 0.0005 μg/mL, respectively (Figure 5A). Moreover, in normal cells (CCL-81), the cytotoxicity of SMA@mTHPC was largely lowered, and a 10-time higher IC_50_ was observed both with light irradiation (IC_50_ of 0.15 μg/mL) and without irradiation (IC_50_ of 20 μg/mL) (Figure 5B).

### 3.5. Intracellular Uptake of SMA@mTHPC

**Figure 6:**
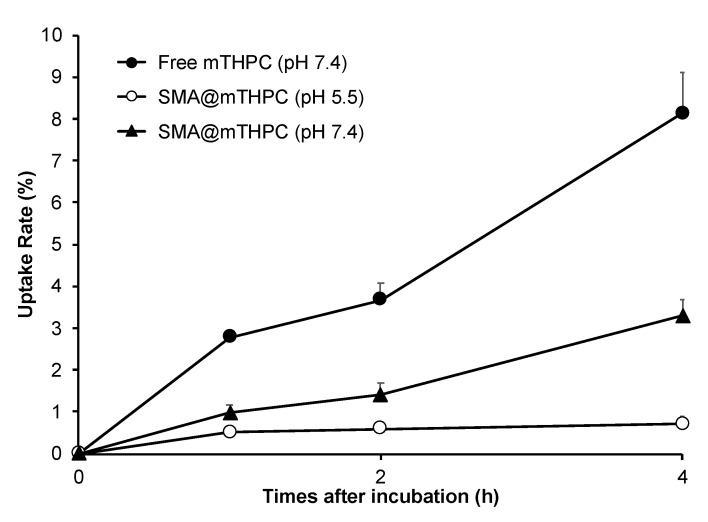
Intracellular uptake of SMA@mTHPC and free mTHPC in mouse colon cancer C26 cells. C26 cells were incubated with SMA@mTHPC or free mTHPC using media of different pH values (7.4 or 5.5). After indicated time, cells were collected, and the internalized mTHPC was quantified by measuring fluorescence intensity. Data are mean ± SD, *n* = 3. See text for details.

As shown in Figure 6, free mTHPC was rapidly taken up by cancer cells, and almost 10% of the applied drugs were internalized within 4 h. In contrast, SMA@mTHPC showed a 10-time lower intracellular uptake than free mTHPC; however, at pH 5.5, the internalization of SMA@mTHPC was significantly increased (Figure 6). These findings are parallel with the results of release profiles (Figure 3), again suggesting the release of free mTHPC is a key factor for the therapeutic effect of SMA@mTHPC.

### 3.6. Tissue Distribution of SMA@mTHPC and In Vivo Imaging

**Figure 7:**
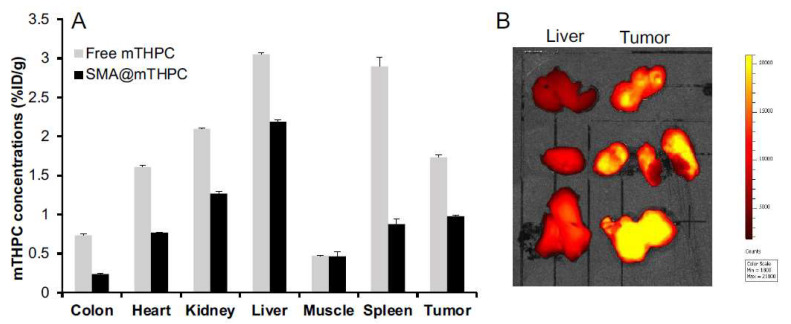
In vivo tissue distribution of SMA@mTHPC at 24 h after i.v. injection in sarcoma S180 tumor-bearing mice. In the S180 solid tumor model, SMA@mTHPC (5 mg/kg mTHPC equivalent) or free mTHPC (5 mg/kg) was i.v. injected when the tumor grew to the size of about 10 mm in diameter; the mice were then killed and indicated tissues were collected, and the amount of SMA@mTHPC in each tissue was quantified by detecting the fluorescence of mTHPC (**A**). In a separate experiment, the tumor and liver of SMA@mTHPC-treated mice were resected and applied to in vivo imaging using IVIS (**B**). See text for details. Data are mean ± SD, *n* = 3–6.

For investigating the body distribution of SMA@mTHPC, we first carried out in vivo imaging in a S180 transplanted tumor model by taking advantage of the fluorescence property of mTHPC. As shown in Figure 7A, we found a relatively high accumulation of SMA@mTHPC in tumor at 24 h after i.v. injection, which was higher than those in most normal tissues including the muscle, colon, and heart. Compared to the tumor, higher accumulation in the liver, which is rich in the reticuloendothelial system that captures macromolecules, was observed (Figure 7A); however, in vivo imaging showed a much lower fluorescence in the liver than that in the tumor (Figure 7B). Moreover, relatively high accumulation was also observed in the kidney, which indicates the gradual release of free mTHPC in circulation. Similar distribution was also found for free mTHPC after i.v. injection, although it had to be dissolved in organic solvent (i.e., DMSO), which suggests that mTHPC may bind to serum proteins, thus behaving as large molecules similar to Evans blue, as described in many previous studies in the literature [6,31].

### 3.7. In Vivo Antitumor PDT Effect of SMA@mTHPC

To investigate the therapeutic (PDT) potential of SMA@mTHPC, we performed in vivo experiments using mouse sarcoma S180 solid tumor model. Treatment was carried out when the tumor grew to about 1 cm in diameter; SMA@mTHPC was first injected i.v., and then light irradiation was performed using xenon light source with a broadband light of 400–700 nm (90 mW/cm^2^, 5 min [27 J/cm^2^]) at 24 and 48 h after injection when SMA@mTHPC accumulated in the tumor preferentially with low accumulation in normal tissues. 

**Figure 8:**
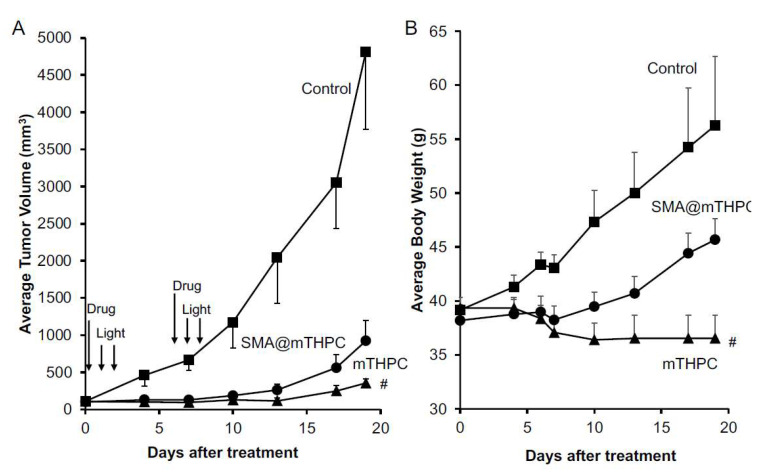
In vivo PDT effect of SMA@mTHPC in S180 solid tumor. A xenon light source was used (MAX-303; Asahi Spectra). Indicated concentrations of SMA@mTHPC were injected i.v. when tumor diameters reached 8–10 mm. After 24 and 48 h, light irradiation (90 mW/cm^2^, 5 min, 27 J/cm^2^) was carried out. Tumor volume (**A**) and body weight (**B**) were measured every 2 or 3 days. Arrows indicate the application of SMA@mTHPC or mTHPC, and light irradiation. Data are means ± SD; *n* = 4–8. # one mouse died during the treatment using mTHPC. See text for details.

The results, as indicated in Figure 8A, showed a significant suppression of tumor growth by the treatment, and similar therapeutic effects were achieved by using either SMA@mTHPC or free mTHPC (Figure 8A). However, during the treatment, we found a significant loss of body weight in free mTHPC-treated mice, in which one mouse died after 3 days of treatment (Figure 8B), whereas PDT using SMA@mTHPC did not show any apparent side effect during the period of observation, and the body weights of mice increased normally similar to control mice without treatment (the difference of body weight between SMA@mTHPC and control is considered mostly due to the difference of tumor weight) (Figure 8B). Free mTHPC-treated mice showed reddish and blackish coloration in the skin around the tumor (Figure S4), indicating severe inflammation in the skin, whereas no apparent changes were found in SMA@mTHPC-treated mice. These findings suggested the superior safety of SMA@mTHPC.

In addition, light irradiation alone or SMA@mTHPC/mTHPC alone without light irradiation did not exhibit apparent tumor growth suppression (Figure S5), indicating that the therapeutic effect was mostly the outcome of PDT.

### 3.8. Decreased Toxicity of SMA@mTHPC Compared to Free mTHPC

Given that SMA@mTHPC exhibited a better safety profile than free mTHPC, as indicated in Figure 8B, we further investigated and compared the toxicity profile of SMA@mTHPC with that of free mTHPC by using different doses and administration routes.

**Figure 9:**
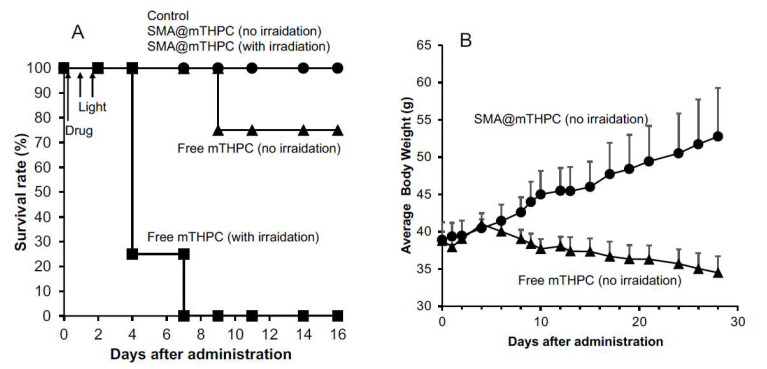
Toxicity profiles of SMA@mTHPC and free mTHPC as indicated by survival rate and body weight change in S180 tumor-bearing mice. (**A**) SMA@mTHPC or free mTHPC was injected i.p. at a concentration of 10 mg/kg (mTHPC equivalent), and in some mice, light irradiation was carried out as described in Figure 7, where the survival rate was recorded. In a separate study, SMA@mTHPC or free mTHPC was injected i.v. at a concentration of 20 mg/kg (mTHPC equivalent) with light irradiation, and the body weight change (**B**) was measured and calculated. Data are means ± SD; *n* = 4–8. See text for details.

First, we administered the drugs to S180 tumor-bearing mice by i.p. route because i.v. route is not a common route for organic solution of mTHPC, and we found 10 mg/kg of SMA@mTHPC did not show any apparent side effects and all mice survived up to 16 days after the treatment, either with light irradiation or without light irradiation (Figure 9A); however, administration of free mTHPC (10 mg/kg, mTHPC equivalent) showed severe toxicity, one out of four mice died even without light irradiation, and all mice died after light irradiation within 1 week after treatment (Figure 9A). Then, we confirmed the toxicity profiles of SMA@mTHPC/free mTHPC alone without light irradiation (but the mice were subjected to ambient light), by i.v. route in which free mTHPC was first dissolved in DMSO at a high concentration and then diluted to experimental concentration by PBS. As shown in Figure 9B, at the dose of 20 mg/kg (mTHPC equivalent), no apparent side effect (body weight loss) was observed for SMA@mTHPC, and all mice survived for up to 28 days after administration. In contrast, administration of free mTHPC induced a remarkable loss of body weight (Figure 9B); the mice were very weak during the experiment period, and one mouse died 9 days after injection. These finding further indicated the superior safety profile of SMA@mTHPC to free mTHPC for PDT.

## 4. Discussion

In the present study, we developed a polymeric micelle of PDT drug mTHPC, SMA@mTHPC, which showed potent therapeutic effect and high safety. SMA@mTHPC micelle was formed by self-assembly in aqueous solution through hydrophobic interaction between mTHPC and hydrophobic moiety of SMA (Figure 1A), with a hydrodynamic size of 98 nm (Figure 1B). As the micelle formation in physiological solution, SMA@mTHPC showed prolonged circulation time and tumor-targeted accumulation based on the EPR effect (Figure 7). SMA@mTHPC micelle is relatively stable in circulation, which ensured its safety because generation of ^1^O_2_ and fluorescence will be quenched in micellar state (Figure 2 and Figure 4). However, the micellar state will be disrupted in the tumor environment and during internalization, resulting in the appearance of strong fluorescence (Figure 7) and generation of ^1^O_2_ (Figure 4). Consequently, potent antitumor PDT effect was achieved with little damage to the host (Figure 8 and Figure 9).

Stability is one important issue for micellar drugs, as unstable micelles will release free drugs in circulation before accumulating in tumors, thus behaving similarly to free small molecular drugs. However, a too stable micelle is also not preferable, because release of active drugs from a micelle that is too slow and too little will largely affect the therapeutic effect. Accordingly, an ideal micelle drug is stable in circulation to achieve EPR effect-based tumor accumulation but rapidly release active drugs in tumor tissues to fulfill the antitumor effect, i.e., tumor environment-responsive nanomedicine. In this context, SMA@mTHPC showed a relatively high stability in physiological solution and in the presence of serum, as evidenced by almost completely fluorescence quenching (Figure 2B,C), as well as no or very little generation of ^1^O_2_ (Figure 4). In vivo imaging also showed relatively low fluorescence intensity in the liver (Figure 7B), although the amount of SMA@mTHPC was relatively high (Figure 7A), which further supported this notion. More importantly and interestingly, tumor showed a strong fluorescence intensity, although tumor concentration was lower than that in the liver (Figure 7). These findings suggested that the micelles are disrupted in tumor tissues, and release of free mTHPC thus exhibits strong fluorescence. The release profile of SMA@mTHPC also indicated the tumor environment responsive behavior of SMA@mTHPC, for which more release occurred at weak acidic pH (6.5) that is seen in most solid tumors [30] than neutral pH (7.4) (Figure 3). Further, when SMA@mTHPC is taken up by tumor cells, in the lysosomal compartment (pH 5.0–5.5), extensive and rapid release of free mTHPC will occur, as indicated by the release profile (Figure 3) and enhanced intracellular uptake (Figure 6) at pH 5.5. It has been reported that SMA-modified nano-drugs showed marked intracellular uptake at acidic pH than at neutral pH because of the higher lipophilicity of maleyl carboxylic at acidic pH (HOOC-COO−) [32,33]. Taken together, the acidic pH of tumor tissue will trigger the release of mTHPC from SMA@mTHPC, as well as intracellular uptake of SMA@mTHPC, which further enhance the release cascade, consequently resulting in the extensive release in tumor tissue.

Benefiting from the tumor environment-responsive behavior as described above, SMA@mTHPC showed a remarkable antitumor PDT effect that was similar to the effect of free mTHPC (Figure 7). More importantly, it ensured the high safety of this treatment. Firstly, as a micellar nano-drug, it accumulated in tumor tissue at a relatively high concentration, whereas it distributes less in most normal tissues (Figure 6). Furthermore, in normal tissues such as the liver and blood, it maintains a stably micellar formation, and thus the generation of ^1^O_2_ is suppressed; consequently, no or very few side effects were observed (Figure 8B and Figure 9). In contrast, although free mTHPC showed a strong antitumor PDT effect, severe side effects including the death of host mice appeared (Figure 8 and Figure 9), and the toxicity of free mTHPC could also be seen even under ambient light (without light irradiation using therapeutic xenon light source) (Figure 9). Indiscriminate tissue distribution and photoexcitation/generation of ^1^O_2_ are the major causes of the toxicity of free mTHPC. In this regard, micellar modification of mTHPC not only decreases the distribution in most normal tissues, but also lowers the photosensitivity in normal tissues, resulting superior safety profiles. These findings, together with the high water-solubility of SMA@mTHPC, strongly suggest the advantages and applicability of SMA@mTHPC, which warrants further investigations.

Compared with free mTHPC, SMA@mTHPC showed lower cytotoxicity both under light irradiation (photocytoxicity) and without light exposure (dark cytotoxicity) (Figure 5). We considered that the reason is mostly that the micelle formation lowers the photosensitivity of mTHPC as described above. This result is also associated to the decreased toxicity of SMA@mTHPC. Moreover, regarding the dark cytoxicitiy of SMA@mTHPC and mTHPC, it may be partly due to the ambient light during incubation; however, mTHPC itself may also has inherent cytotoxicity, although the mechanism is not elucidated. Therefore, further studies are needed to clarify this issue.

Regarding the tissue distribution of SMA@mTHPC, besides the relatively high tumor accumulation, SMA@mTHPC also accumulates at high levels in the liver (Figure 7A). However, the fluorescence imaging exhibited a relatively low signal (Figure 7B). This finding is most probably due to the micellar stability in the liver as the polymer stays in a blood-like environment. The high concentrations of heme in the liver that may absorb light near the absorption band of mTHPC may also suppress the fluorescence of mTHPC. Taken together, these findings again support environment-responsive behavior of SMA@mTHPC: it is stable as a micelle in circulation and normal tissues such as those in the liver, thus showing very little toxicity and adverse effects (Figure 7 and Figure 8), whereas micelle formation is destroyed in the tumor environment, thus exhibiting remarkable tumor imaging potential (Figure 7) as well as antitumor PDT effect (Figure 8). As for free mTHPC, we also found a relative tumor concentration that was similar to SMA@mTHPC (Figure 6). This was probably due to its binding property to circulation proteins such as albumin, as seen in Evans blue, the commonly used agent to indicate EPR effect that is known to bind to albumin and thus behave as a macromolecule. Similar behaviors have also been observed in some anticancer drugs such as paclitaxel and gemcitabine [34,35]. Thus, in circulation, mTHPC may behave as a macromolecule (albumin complex) showing prolonged retention, which was slightly higher than that of SMA@mTHPC (Figure 7). However, this complex formation of mTHPC is not well organized and the fluorescence and phototoxicity of mTHPC could not be efficiently suppressed by this formation; thus, although mTHPC showed a potent antitumor PDT effect, its long circulation time due to its protein binding, in contrast, induced severe side effects (Figure 8B and Figure 9).

Taken together, these findings suggested SMA@mTHPC could become a candidate drug for PDT, which not only shows potent therapeutic effect, but more importantly exhibits high safety profiles, and thus we anticipate its application in the future.

## Data Availability

The data presented in this study are available on request from the corresponding author.

## Supplementary Materials

The following are available online at https://www.mdpi.com/article/10.3390/biomedicines9101493/s1, Figure S1: UV–VIS spectra of SMA@mTHPC and free mTHPC, Figure S2: Standard curve of mTHPC in DMSO as measured by fluorescence (Ex420nm/Em590nm), Figure S3: Pictures of SMA@mTHPC solution in PBS, Figure S4: Pictures of mice after free mTHPC treatment and SMA@mTHPC treatment, Figure S5: In vivo antitumor effect of irradiation alone (without SMA@mTHPC) and SMA@mTHPC alone (without light irradiation).

## References

[1] McBride G. (2002). Studies expand potential uses of photodynamic therapy. J. Natl. Cancer Inst..

[2] Wilson B.C. (2002). Photodynamic therapy for cancer: Principles. Can. J. Gastroenterol..

[3] Dolmans D.E., Fukumura D. (2003). Photodynamic therapy for cancer. Nat. Rev. Cancer.

[4] O’Connor W., Gallagher A. (2009). Byrne, Porphyrin and nonporphyrin photosensitizers in oncology: Preclinical and clinical advances in photodynamic therapy. Photochem. Photobiol..

[5] Fang J., Seki T. (2009). Therapeutic strategies by modulating oxygen stress in cancer and inflammation. Adv. Drug Deliv. Rev..

[6] Matsumura Y., Maeda H. (1986). A new concept for macromolecular therapeutics in cancer chemotherapy: Mechanism of tumoritropic accumulation of proteins and the antitumor agent SMANCS. Cancer Res..

[7] Torchilin V. (2011). Tumor delivery of macromolecular drugs based on the EPR effect. Adv. Drug Deliv. Rev..

[8] Fang J., Nakamura H. (2011). The EPR effect: Unique features of tumor blood vessels for drug delivery, factors involved, and limitations and augmentation of the effect. Adv. Drug Deliv. Rev..

[9] Maeda H. (2015). Toward a full understanding of the EPR effect in primary and metastatic tumors as well as issues related to its heterogeneity. Adv. Drug Deliv. Rev..

[10] Fang J., Islam W. (2020). Exploiting the dynamics of the EPR effect and strategies to improve the therapeutic effects of nanomedicines by using EPR effect enhancers. Adv. Drug Deliv. Rev..

[11] Islam R., Maeda H. (2021). Factors affecting the dynamics and heterogeneity of the EPR effect: Pathophysiological and pathoanatomic features, drug formulations and physicochemical factors. Expert Opin. Drug Deliv..

[12] Nakamura H., Liao L. (2013). Micelles of zinc protoporphyrin conjugated to N-(2-hydroxypropyl) me- thacrylamide (HPMA) copolymer for imaging and light-induced antitumor effects in vivo. J. Control Release.

[13] Islam W., Matsumoto Y. (2021). Polymer-conjugated glucosamine complexed with boric acid shows tumor-selective accumulation and simultaneous inhibition of glycolysis. Biomaterials.

[14] Fang J., Liao L. (2015). Photodynamic therapy and imaging based on tumor-targeted nanoprobe, polymer-conjugated zinc protoporphyrin. Future Sci. OA.

[15] Fang J., Islam W. (2018). N-(2-hydroxypropyl) methacrylamide polymer conjugated pyropheophorbide-a, a promising tumor-targeted theranostic probe for photodynamic therapy and imaging. Eur. J. Pharm. Biopharm..

[16] Fang J., Tsukigawa K. (2016). Styrene-maleic acid-copolymer conjugated zinc protoporphyrin as a candidate drug for tumor-targeted therapy and imaging. J. Drug Target.

[17] Iyer A.K., Greish K. (2007). Polymeric micelles of zinc protoporphyrin for tumor targeted delivery based on EPR effect and singlet oxygen generation. J. Drug Target.

[18] Iyer A.K., Greish K. (2007). High-loading nanosized micelles of copoly (styrene-maleic acid)-zinc protoporphyrin for targeted delivery of a potent heme oxygenase inhibitor. Biomaterials.

[19] Fang J., Sawa T. (2003). In vivo antitumor activity of pegylated zinc protoporphyrin: Targeted inhibition of heme oxygenase in solid tumor. Cancer Res.

[20] Sahoo S.K., Sawa T. (2002). Pegylated zinc protoporphyrin: A water-soluble heme oxygenase inhibitor with tumor-targeting capacity. Bioconjug. Chem..

[21] Dragicevic-Curic N., Scheglmann D. (2009). Development of different temoporfin-loaded invasomes-novel nanocarriers of temoporfin: Characterization, stability and in vitro skin penetration studies. Colloids Surf. B Biointerfaces.

[22] Dragicevic-Curic N., Fahr A. (2012). Liposomes in topical photodynamic therapy. Expert Opin. Drug Deliv..

[23] Yakavets I., Francois A. (2021). Effect of stroma on the behavior of temoporfin-loaded lipid nanovesicles inside the stroma-rich head and neck carcinoma spheroids. J. Nanobiotechnology.

[24] Yakavets I., Millard M. (2019). Current state of the nanoscale delivery systems for temoporfin-based photodynamic therapy: Advanced delivery strategies. J. Control Release.

[25] Cabral H., Miyata K. (2018). Block Copolymer Micelles in Nanomedicine Applications. Chem. Rev..

[26] Cabral H., Kataoka K. (2014). Progress of drug-loaded polymeric micelles into clinical studies. J. Control Release.

[27] Greish K., Sawa T. (2004). SMA-doxorubicin, a new polymeric micellar drug for effective targeting to solid tumours. J. Control Release.

[28] Greish K., Nagamitsu A. (2005). Copoly (styrene-maleic acid)-pirarubicin micelles: High tumor-targeting efficiency with little toxicity. Bioconjug. Chem..

[29] Li Q., Li Z. (2017). The Strong Light-Emission Materials in the Aggregated State: What Happens from a Single Molecule to the Collective Group. Adv. Sci. (Weinh).

[30] Racker E. (1972). Bioenergetics and the problem of tumor growth. Am. Sci..

[31] Fang J., Qin H. (2012). Carbon monoxide, generated by heme oxygenase -1, mediates the enhanced permeability and retention effect in solid tumors. Cancer Sci..

[32] Oda T., Maeda H. (1987). Binding to and internalization by cultured cells of neocarzinostatin and enhancement of its actions by conjugation with lipophilic styrene-maleic acid copolymer1. Cancer Res..

[33] Maeda H., Islam W., Pasut G., Zalipsky S. (2020). Overcoming barriers for tumor-targeted drug delivery: The power of macromolecular anticancer drugs with the EPR effect and the modulation of vascular physiology. Polymer-Protein Conjugates: From Pegylation and Beyond.

[34] Qi H., Wang Y. (2021). The different interactions of two anticancer drugs with bovine serum albumin based on multi-spectrum method combined with molecular dynamics simulations. Spectrochim. Acta A Mol. Biomol. Spectrosc..

[35] Ali M.S., Muthukumaran J. (2021). Experimental and computational investigation on the binding of anticancer drug gemcitabine with bovine serum albumin. J. Biomol. Struct. Dyn..

