# Supplementary material for: Styrene Maleic Acid Copolymer-Based Micellar Formation of Temoporfin (SMA@ mTHPC) Behaves as A Nanoprobe for Tumor-Targeted Photodynamic Therapy with A Superior Safety
Source: Biomedicines. 2021 Oct 19;9(10):1493. doi: 10.3390/biomedicines9101493 (PMC8533298; doi:10.3390/biomedicines9101493)
Supplement: Supplementary file 1 [file biomedicines-09-01493-s001.zip › biomedicines-1380282-supplementary.pdf]

Supplementary data Figure S1

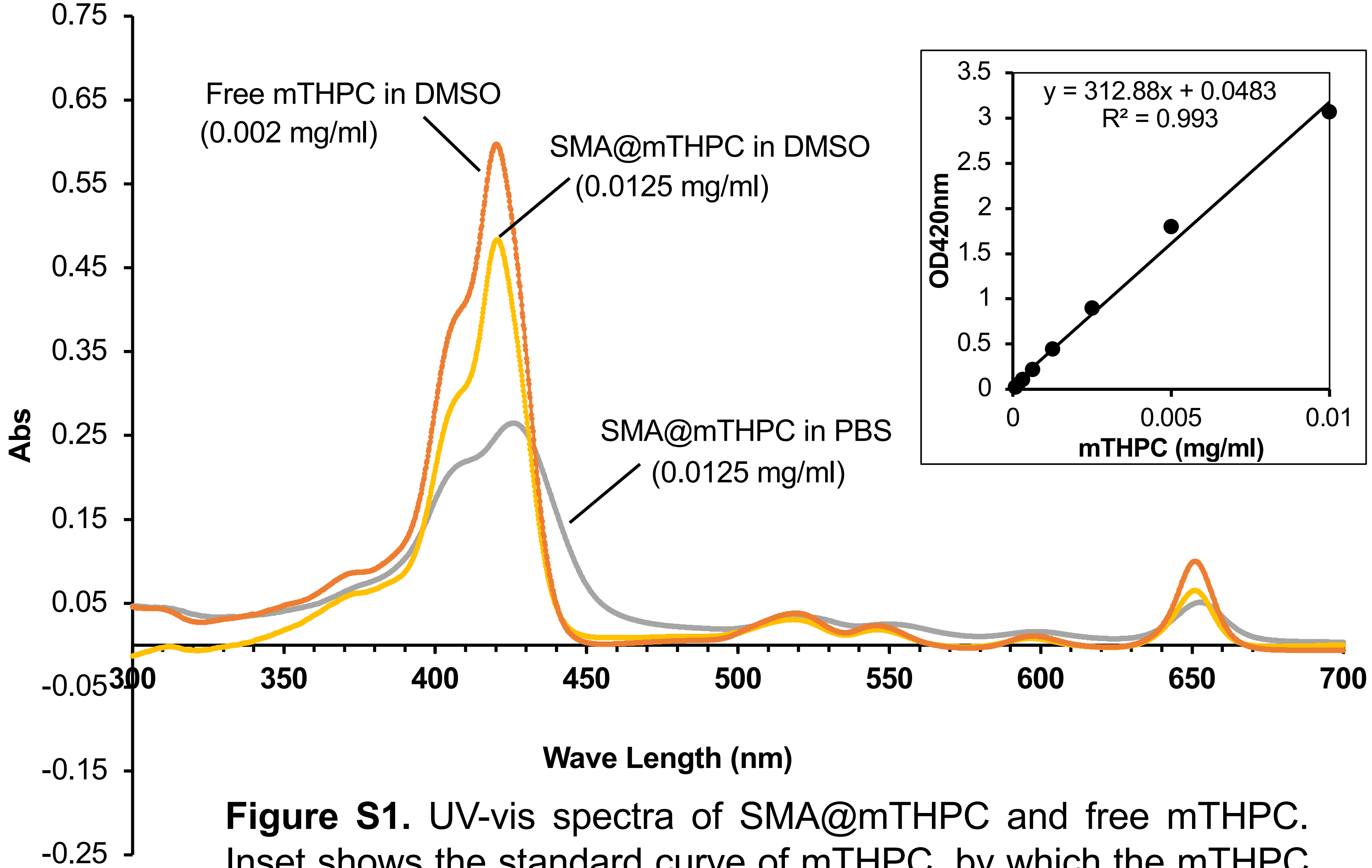

Figure S1. UV-vis spectra of SMA@mTHPC and free mTHPC. Inset shows the standard curve of mTHPC, by which the mTHPC loading in SMA@mTHPC was calculated as 10%.

Supplementary data Figure S2

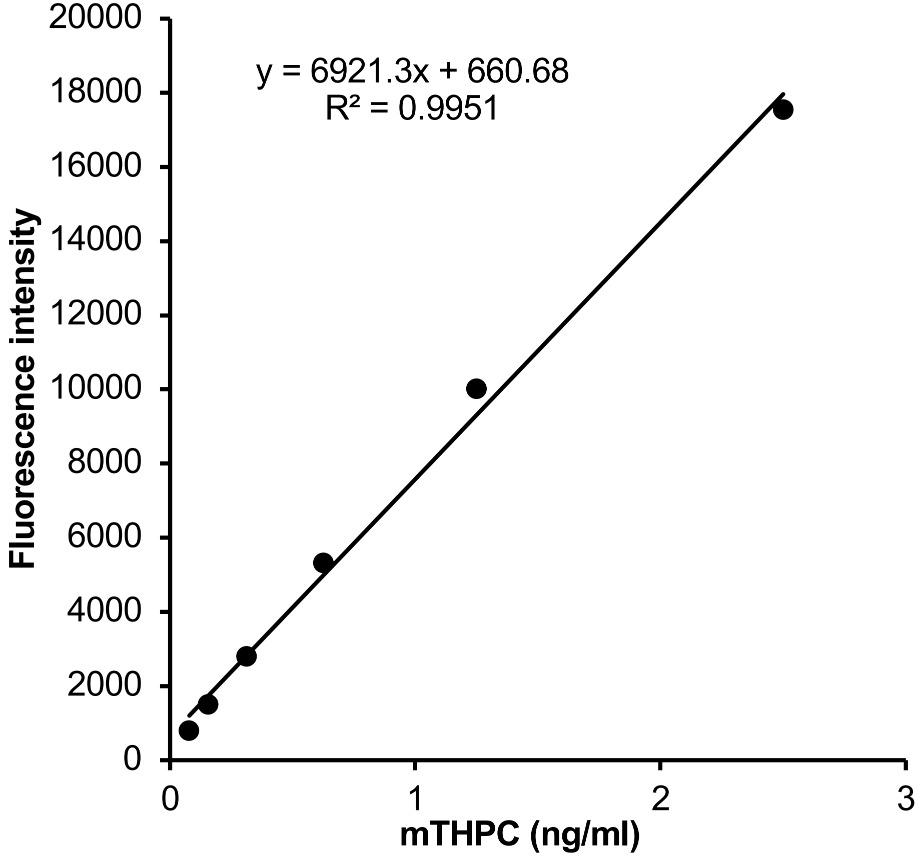

Figure S2. Standard curve of mTHPC in DMSO as measured by fluorescence (Ex420nm/Em590nm).

Supplementary data Figure S3

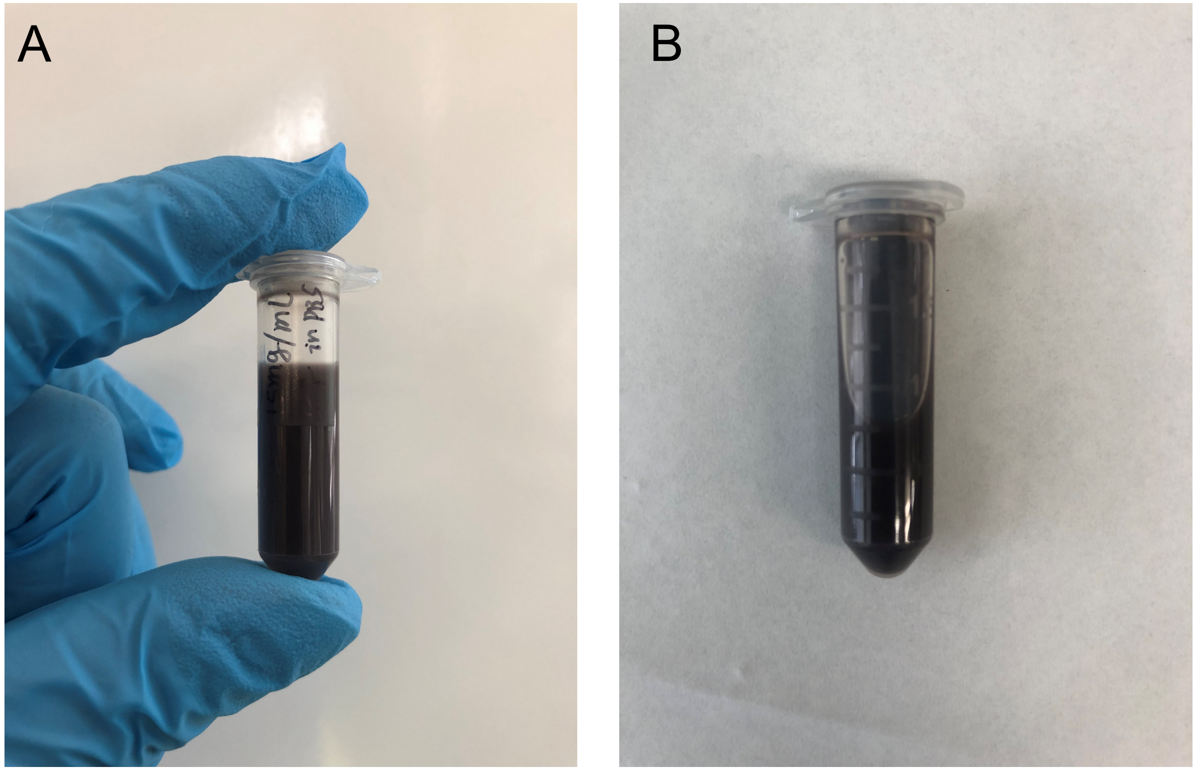

Figure S3. Pictures of SMA@mTHPC solution in PBS at 20 mg/ml before (A) and after centrigugation (12000 rpm, 1 min) (B).

Supplementary data Figure S4

Free mTHPC-treated mice SMA@mTHPC-treated mice

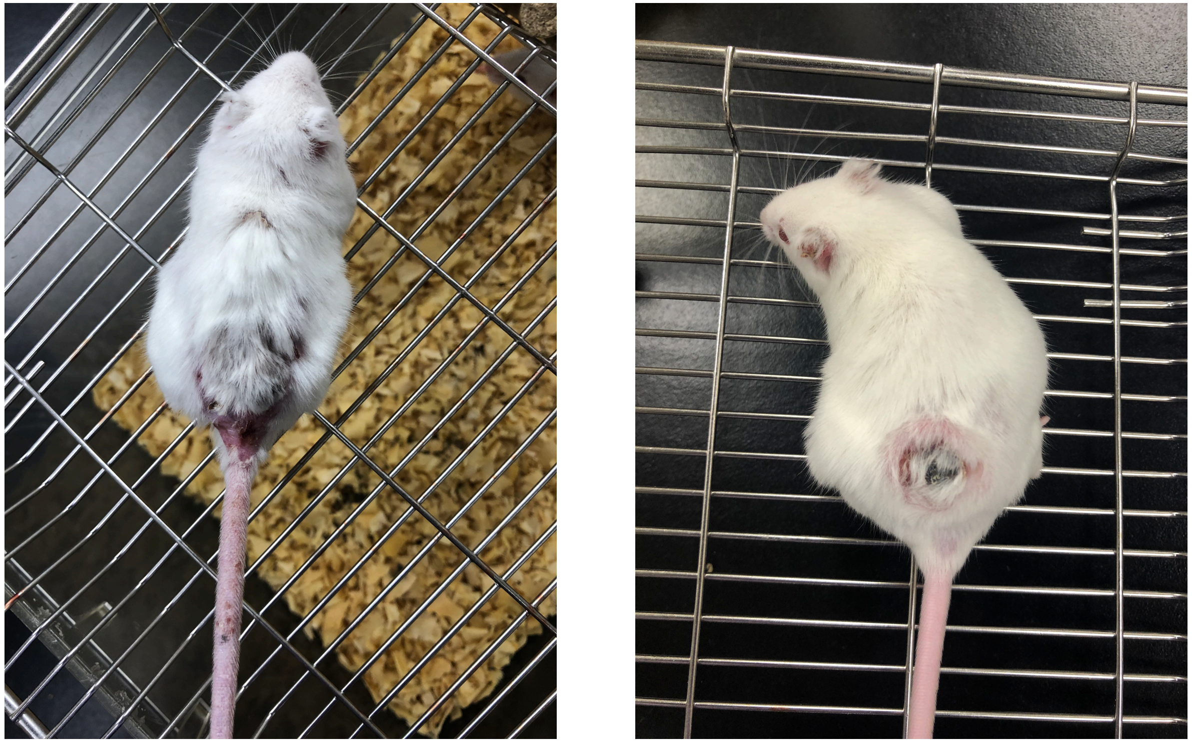

Figure S4. Pictures of mice after free mTHPC treatment and SMA@mTHPC treatment.

Supplementary data Figure S5

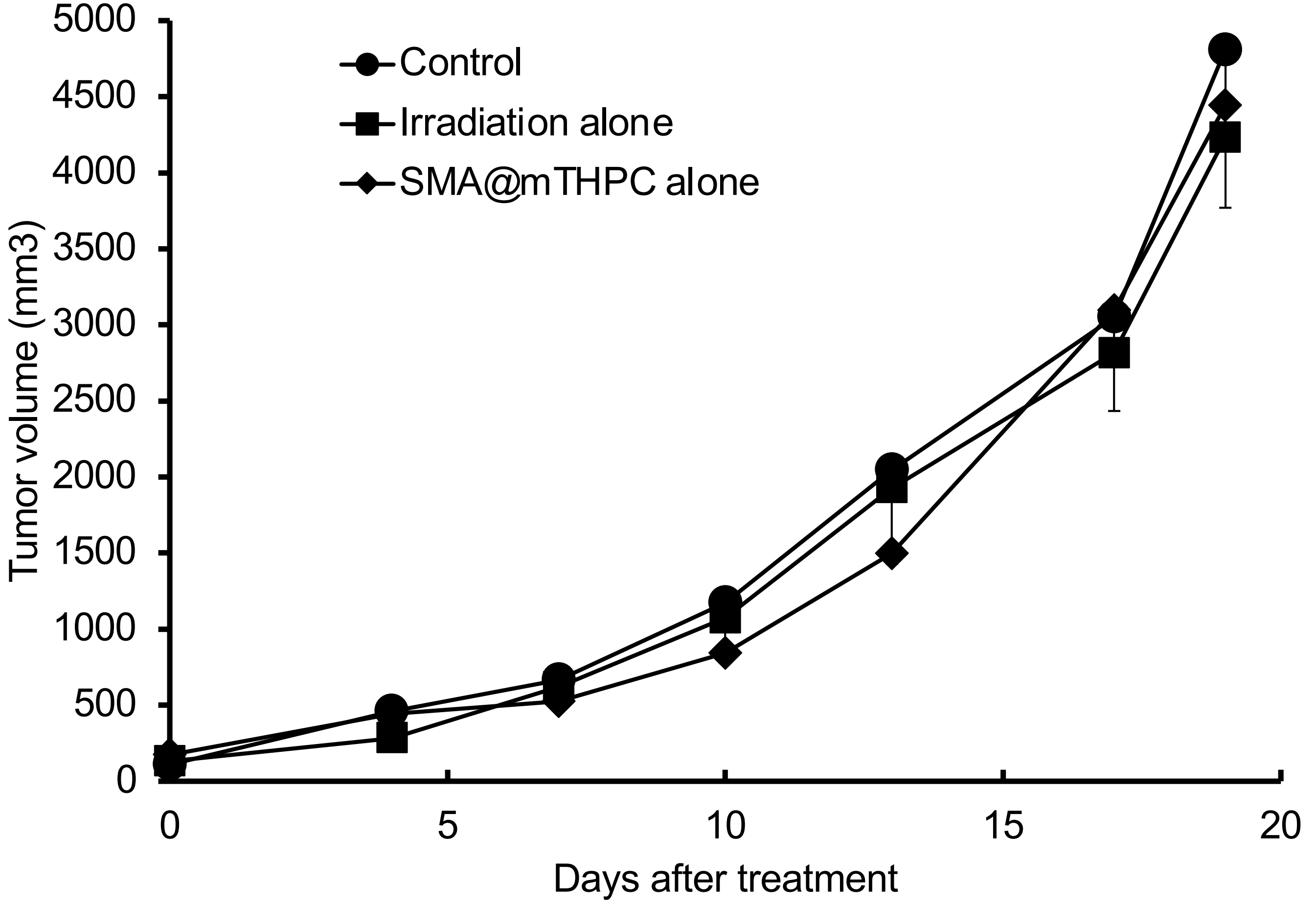

Figure S5. *In vivo* antitumor effect of irradiation alone (without SMA@mTHPC) and SMA@mTHPC alone (without light irradiation).
